# *Myriad* and its implications for patent protection of isolated natural products in the United States

**DOI:** 10.1186/1749-8546-9-17

**Published:** 2014-07-01

**Authors:** Alice Yuen-Ting Wong, Albert Wai-Kit Chan

**Affiliations:** 1 Albert Wai-Kit Chan Intellectual Property Limited, Hong Kong, SAR, China; 2 Law Offices of Albert Wai-Kit Chan, PLLC, Whitestone, NY 11357, USA

## Abstract

Extracts and compounds of natural products have potential as alternatives to current Western medicines. However, these products may not be patentable under the statutory requirements because of their naturally-occurring nature. This article analyzes the current patenting practices for natural products in the United States, particularly in light of the recent Supreme Court ruling in *Myriad*, and suggests an advantageous strategy for patenting these products. Briefly, isolated natural products per se are not patentable in the United States. Therefore, patenting focus should be placed on the modification, formulation, manufacture, and application of natural products. A detailed description of each invention is highly recommended for stronger support and broader coverage of the claims.

## Background

Some natural products have gained popularity in pharmaceutical and healthcare industries for their therapeutic potential. Numerous extracts and compounds isolated from natural products are included in drugs and healthcare supplements. For instance, a supplement of red clover isoflavones and a tablet of soy isoflavones were used to reduce hot flushes in women [1,2], and extracts of valerian, hops, and passion flower were blended into a sleep aid tablet to improve sleep [3].

Patents offer exclusive rights to protect innovations and achievements, and can attract investment to sustain and strengthen further research. Chinese medicine research and development substantially relies on preparing extracts and compounds from natural materials, screening for bioactive compounds effective for particular conditions or diseases, and formulating these products into drugs and supplements. Patent protection for these extracts and compounds is desired. The patenting policy on natural products was recently revisited in the United States in light of the recent Supreme Court ruling in *Myriad*, which held that an isolated DNA molecule from the human breast cancer-susceptibility gene *BRCA* was patent-ineligible [4].

This article analyzes the current patenting practices for natural products in the United States, particularly in light of *Myriad*, and provides suggestions and advice toward a more advantageous strategy for patenting these products.

### Are natural products patentable?

An invention must be new and useful to be patentable in the United States [5]. Notably, laws of nature, natural phenomena, and abstract ideas are excluded from patent-eligible protection [6], because these naturally-occurring matters are discovered rather than invented. Natural products would not be awarded a patent merely for their discovery. Patent eligibility is essentially different from patentability. Patent eligibility means that the invention is an item that can seek a protection under patent law. In contrast, an invention becomes patentable when it meets the necessary requirements, such as novelty, inventiveness, and industrial applicability, and a patent can eventually be granted as well. Hence, a non-natural compound is patent-eligible, but becomes unpatentable if it is known.

The patent eligibility of natural products isolated or purified from natural materials is less affirmatively defined. The US patent law protects new, useful, and non-obvious chemical compounds and compositions [7], encompassing isolated and purified products from natural materials. It has been intensively discussed in the Courts whether a product isolated from natural materials is patent-eligible or represents another kind of substance that should be exempted from the "product of nature" doctrine. However, the Courts adopt inconstant grounds and standards for determining the patent eligibility of isolated natural products, which leaves doubt about exactly how different an isolated natural product needs to be from its natural counterpart to be sufficient for exemption [8].

The Courts acknowledged that "even if [products] were merely an extracted product without change, there is no rule that such products are not patentable" [9]. However, an isolated product would not be patentable for its improved purity, unless it results in "properties and characteristics which were different in kind from those of the known product rather than in degree" [10]. A recrystallized aspirin and a purified form of adrenalin were deemed patentable because the purer forms were therapeutically different from the natural forms [9,11]. In another case, vitamin B_12_ purified from the fermentation of fungi was considered to be of great therapeutic and commercial worth [12]. Similarly in *Chakrabarty*, the Courts held that a genetically engineered bacterium capable of breaking down crude oil was patentable because the bacterium had "markedly different characteristics from any found in nature" [13]. In the above cases, the Courts took the view that the isolation or purification had transformed the natural product to another "kind" of substance that was functionally different from the natural product.

On the contrary, purified tungsten from tungsten ore and a naturally-occurring bacterial strain were held to be unpatentable because the Courts recognized that the commercial usefulness of the purified tungsten and bacteria could be attributed to their intrinsic properties [14,15]. In these cases, the patentees had simply discovered "the natural qualities of the pure tungsten" [14] and did not create "a state of inhibition or of non-inhibition in the bacteria" [15]. The Courts adopted an opposite view that a functional difference was insufficient for transforming a natural product to another "kind" of substance.

An isolated product must be significantly distinct from the natural form in a certain way to be patentable, regardless of the inconsistent rulings in the above cases. However, in the absence of robust standards for determining whether a difference is "in kind" or "in degree", or whether it is a "marked difference", the patentability of isolated natural products and the enforceability of patents for these products remain ambiguous.

Recently, the "product of nature" doctrine was revisited in *Myriad*, which invalidated a patent on an isolated DNA molecule from the human cancer-susceptibility gene *BRCA*[4]. The Supreme Court affirmed that an isolated DNA molecule with an identical sequence to a natural gene is a naturally-occurring product and not patentable. On the other hand, a complementary DNA (cDNA) of *BRCA* that had an intron removed was patentable because it is not naturally-occurring. However, a short strand of cDNA that is indistinguishable from natural DNA may not be patentable.

In *Myriad*, the Supreme Court recognized that the patentee had isolated a DNA molecule from the human genome by severing the chemical bonds between nucleotides. Although the isolated DNA molecule is not naturally-occurring in any human body, the Supreme Court disagreed that the isolation constituted an act of invention and stated that the isolated DNA had no "markedly different characteristics from any found in nature" [4]. The Supreme Court also declined to agree that the severing had created a non-naturally-occurring molecule because the claims were not expressed in terms of the chemical composition of the DNA but primarily concerned the genetic information in the nucleotide sequence, which was not created by the patentee.

In view of *Chakrabarty*[13], *Myriad*[4], and *Mayo*[16] (in which the Courts invalidated diagnostic claims that merely recited natural principles), the US patent office (USPTO) revisited the patenting policy and issued a comprehensive examination guideline for natural matters on 4 March 2014 [17]. The guideline provides a list of factors weighting for and against the patent eligibility of claims reciting or involving laws of nature/natural principles, natural phenomena, and natural products [17].

Under this guideline, the patent eligibility of a claim is principally determined by considering whether the claim recites something that is "significantly" or "markedly" different from what exists, provided that not all differences rise to the level of "marked differences" and that "a marked difference must be a significant difference, *i.e.*, more than an incidental or trivial difference" [17].

A functional difference, such as a new utility, is not a mandatory weighting factor. Words such as "isolated", "recombinant", and "synthetic" add no weight to the patent eligibility. However, a structural difference is a primary, but not sole, criterion for patent eligibility. As seen in *Myriad*[4], the structural difference of an isolated gene from the chromosomal counterpart is not regarded as a "marked" difference and is therefore patent-ineligible (page 5, line 11 of [17]). In contrast, the guideline determines that a synthetic derivative of natural amazonic acid, 5-methyl amazonic acid, has a "marked" structural difference from the natural acid and is therefore patent-eligible (page 8, line 10 of [17]). Accordingly, a structural difference appears to be important, but not necessarily sufficient, for patent eligibility. When a structural difference is absent or insignificant, a functional difference may also be considered. A structural difference is deemed sufficient for patent eligibility, whereas a functional difference may or may not be considered in chemical products. In the absence of a structural difference, a natural product must exhibit new functions or characteristics that rise to the level of "marked" differences to be patent-eligible. Accordingly, amazonic acid purified from the leaves of the Amazonian cherry tree is not significantly different from the naturally-occurring amazonic acid, and hence falls within the "product of nature" doctrine. On the contrary, a synthetic derivative of amazonic acid, 5-methyl amazonic acid, is patent-eligible because it is structurally different from the natural acid. Similarly, a product obtained from a particular manufacturing process in a "product-by-process" claim cannot survive if it has no structural or "marked" difference from the natural form [17].

The same standard applies to the composition of multiple natural products, that is, the composition remains a product of nature if the combined products show no marked difference from the natural products. However, manufactures or compositions of natural products may be patent-eligible if they include additional element(s) that would make the products "something significantly different than the natural products by themselves" [17].

A method of using natural products should involve a practical, specific, and significant application of the natural products such that the claimed method would not foreclose others from using the same natural product in other ways [17]. That is, the method should not merely recite: (i) a general instruction to apply or use the natural products; (ii) well-understood, purely conventional, or routine steps in the relevant field; or (iii) steps that need to be used by anyone who applies or uses the natural products. For instance, a method for treating a disease using a natural product should include additional step(s), such as a regimen and dosage for administration of the natural product used in the treatment, to limit the scope of the claim. Accordingly, a method for treating colon cancer by administering amazonic acid to a patient may not survive as a significant application, whereas a method for treating colon cancer by administering amazonic acid to a patient for 10–20 days at a daily dose of 0.76–1.25 teaspoons would satisfy the requirements [17].

Table 1 summarizes the patent eligibility of different types of natural products and related methods under the new examination policy of the USPTO. Under this policy, extracts and isolated products from natural materials are likely to be unpatentable. A formulation of combined extracts and isolated products may be patentable if the combination results in "markedly different" properties of the combined products, *e.g.*, enhanced efficacy at a lower dosage. Conversely, manufacturing methods such as extraction of natural products and preparation of drugs and supplements using natural products are still patentable if they satisfy other statutory requirements such as novelty and non-obviousness (inventiveness).

**Table 1:** Summary of the patent eligibility of different types of natural products and related methods under the new examination policy of the USPTO

**Products**	**Different from natural product?**		**Patentable subject matter?**	**Examples/Notes**	**References**
	**Structure**	**Characteristics**			
Chemicals in natural materials	No	No	No	Paclitaxel (taxol).	/
Isolated or purified extracts/chemicals	No	No	No	Paclitaxel purified from Pacific yew tree.	Page 7, Example B of [17]
	No	Yes	Maybe	In the absence of a structural difference, the isolated product needs to be combined with something else that leads to a "marked" difference.	
Compositions of natural products	No	No	No	Composition of paclitaxel and hydrogel as a sustained-release and site-specific formulation.	Page 10–11, Example D of [17]
	No	Yes	Maybe	Maybe patentable if the combination attains "marked difference" in properties of the natural products.	
				e.g. A sustained-release and site-specific formulation of paclitaxel and hydrogel at 1:1 ratio with synergistically enhanced efficacy.	
Synthetic natural products	No	No	No	Paclitaxel produced by a total synthesis method.	Page 7, Example B of [17]
Synthetic derivatives of natural products	Yes	No	Yes	Non-naturally-occurring derivative of paclitaxel.	Page 8, Example B of [17]
Compound obtained by a manufacturing process	No	No	No	Paclitaxel purified from Pacific yew tree through a novel purification scheme.	Page 7, Example B of [17]
Method of preparing natural products	No	Yes/No	Yes	Method of extracting paclitaxel from Pacific yew tree, or method of synthesizing paclitaxel.	/
Method of using natural products	No	N/A	Maybe	Patentable if the use is practical and significant.	Page 8–9, Example B of [17]
				e.g. Treating ovarian cancer by administering 175 mg/m^2^ of paclitaxel intravenously over 3 hours every 3 weeks to a patient.	

Accordingly, a proper patenting strategy should focus on derivatives of natural products with enhanced properties, optimized formulations and dosages of natural products, new uses or applications of natural products, and advanced methods for preparing natural products. Claims should be drafted within a reasonable scope and be of sufficient significance so that they do not appear to be a general application of the natural products.

### "Product-by-process" claims

There are circumstances in which a product cannot be "truly" defined by features such as composing ingredients, physical properties, and chemical structure. "Product-by-process" claims define how a product is made, *e.g.*, "product X obtained by process Y" or "a product produced by steps a, b, and c". Sometimes, a "product-by-process" claim is more desirable than a true product claim to cover all possible products produced by a specific manufacturing process, as it may be difficult to identify all possible embodiments and combinations of the products. Most jurisdictions only accept "product-by-process" claims when there are no other possible means and only the manufacturing process can define the products. In the United States, "product-by-process" claims are allowed if the products could not otherwise be adequately defined [18].

In the United States, the patentability of "product-by-process" claims is determined solely on the basis of the product itself, regardless of the manufacturing process. Therefore, a product identical to, or similar to, a known product is not patentable even if it is manufactured by a new or different method [19]. The manufacturing process is taken into account only when the structure of the product is "implied by the process steps" or the "process steps would be expected to impart distinctive structural characteristics to the final product" [20]. If the product claimed is found to be identical or similar to any known products, the applicants would bear the burden of proof on novelty and non-obviousness of the claimed product to demonstrate that the claimed product is distinguishable from the known products [21].

A product isolated or synthesized by a particular process would not be patentable if the patentee cannot prove that the obtained product is non-naturally-occurring and markedly different from the natural products [17]. Paradoxically, the obtained product would be adequately defined if such proof was available, and therefore not permitted to be defined in a "product-by-process" claim.

Enforcement of "product-by-process" claims can be difficult because explicit characteristics of the product are lacking. Notably, a "product-by-process" claim does not necessarily cover all of the equivalent products that are made by other methods, especially if the manufacturing process involves special steps that make the product distinguishable from the known products.

When it comes to infringement, both the product and the manufacturing process of "product-by-process" claims are considered. "Product-by-process" claims only cover products that are produced by the process recited in the claim [22], and therefore an accused product made by a different process does not infringe a "product-by-process" claim [23].

Accordingly, "product-by-process" claims may confer a limited scope of protection and risk invalidation if the patentee cannot prove that no other characterization of the product is possible. Researchers are highly advised to include all features and available information that may be useful, even inadequately, to characterize the products in the application, as well as draft claims based on the information. It is also desirable to draft two sets of claims separately directed toward the manufacturing process (process claim) and the product ("product-by-process" claim) for a broader scope of protection.

### Sufficient disclosures

We usually draft claims reciting a general formula to include a class of molecules sharing common structures or features. A person skilled in the art could deduce various embodiments of the invention. For example, a person skilled in the art would envision that CH_4_, C_3_H_8_, C_100_H_202_, and the like can fall within a claim reciting the general formula (C_n_H_2n+2_). However, contradictory to the common understanding, a claim reciting a general formula is not patentable if only a few species of the general formula have been disclosed in the application, and thereby only confers protection on species that have been explicitly described in the application [24]. The US patent law requires an invention to be sufficiently described in an application such that one skilled in the art would recognize the inventions. That is, if only CH_4_ and C_3_H_8_ have been identified in the application, claims directed toward the general formula (C_n_H_2n+2_) are likely required to be limited to CH_4_ and C_3_H_8_.

A more detailed description would allow one to encompass more embodiments of an invention, and hence achieve a broader scope of protection. It is highly recommended to explore all potential substitutions and modifications of the invention within a reasonable scope, and to disclose as many of these variations as possible in the application. Claims should be drafted to include all enabling and foreseeable embodiments of the invention for subsequent research development and marketing interests of the researchers. Detailed descriptions should not be limited to products, but can also be applied to other technical features such as temperature, pH, and reagents used in the experiments.

## Conclusion

In view of the new examination policy, a patenting strategy should focus on modifications, optimized formulations, manufacturing methods, and specific applications of the natural products. Disclosure of detailed information related to the natural products in the application to comply with the written descriptions and enablement requirements is highly recommended. Identification and characterization of individual embodiments and/or combinations thereof for the invention are necessary for broader coverage of claims. Frequent reviews and revisits of the developments in patenting policy and their patenting strategy will help inventors to better protect their inventions.
